# Low-Velocity Impact Resistance of 3D Re-Entrant Honeycomb Sandwich Structures with CFRP Face Sheets

**DOI:** 10.3390/polym15051092

**Published:** 2023-02-22

**Authors:** Zhen Cui, Jiaqi Qi, Yuechen Duan, Ying Tie, Yanping Zheng, Jun Yang, Cheng Li

**Affiliations:** 1 School of Mechanical and Power Engineering, Zhengzhou University, Zhengzhou 450001, China; 2 School of Aerospace and Mechanical Engineering, Changzhou Institute of Technology, Changzhou 213032, China

**Keywords:** honeycomb structures, impact resistance, low-velocity impact, re-entrant honeycomb, energy absorption

## Abstract

Lightweight sandwich structures have been receiving significant attention. By studying and imitating the structure of biomaterials, its application in the design of sandwich structures has also been found to be feasible. With inspiration from the arrangement of fish scales, a 3D re-entrant honeycomb was designed. In addition, a honeycomb stacking method is proposed. The resultant novel re-entrant honeycomb was utilized as the core of the sandwich structure in order to increase the impact resistance of the sandwich structure under impact loads. The honeycomb core is created using 3D printing. By using low-velocity impact experiments, the mechanical properties of the sandwich structure with Carbon-Fiber-Reinforced Polymer (CFRP) face sheets under different impact energies were studied. To further investigate the effect of the structural parameters on the structural, mechanical properties, a simulation model was developed. Simulation methods examined the effect of structural variables on peak contact force, contact time, and energy absorption. Compared to traditional re-entrant honeycomb, the impact resistance of the improved structure is more significant. Under the same impact energy, the upper face sheet of the re-entrant honeycomb sandwich structure sustains less damage and deformation. The improved structure reduces the upper face sheet damage depth by an average of 12% compared to the traditional structure. In addition, increasing the thickness of the face sheet will enhance the impact resistance of the sandwich panel, but an excessively thick face sheet may decrease the structure’s energy absorption properties. Increasing the concave angle can effectively increase the energy absorption properties of the sandwich structure while preserving its original impact resistance. The research results show the advantages of the re-entrant honeycomb sandwich structure, which has certain significance for the study of the sandwich structure.

## 1. Introduction

With the development of new technologies and materials, research on lightweight structures has attracted a great deal of interest [1]. Among them, lightweight sandwich structures have been rapidly developed because of their excellent mechanical properties, such as superior energy absorption properties, high specific stiffness, and specific strength [2,3]. Due to their manufacturing and performance advantages, sandwich structures are utilized in aerospace and automotive industries as energy-absorbing and protective components [4,5]. Even though sandwich structures have advantages in many fields, research has shown that sandwich structures experience a significant reduction in stiffness and strength under impact loads [6]. Moreover, damage to the sandwich structure caused by low energy is not obvious, which raises the cost of detection. As a result, there has been a lot of focus on studying the impact resistance of sandwich structures.

The typical sandwich structure consists of a face sheet and a core. The materials that make up the face sheet are diverse, including several common isotropic metals and composite materials [7,8,9]. In general, the core of a sandwich structure is a lightweight porous structure, such as foam, honeycomb, or truss [10,11]. Many researchers [12,13,14,15] have contributed to the experimental and theoretical analysis methods of sandwich structures. Gunes [12] established a numerical model to anticipate the low-velocity impact response of aluminum honeycomb sandwich structures and conducted a series of impact tests on sandwich specimens of aluminum honeycomb cores and aluminum panels to improve the accuracy of the numerical simulation model. The impact performance of an aluminum honeycomb sandwich panel is significantly affected by the size of the honeycomb core. With a smaller honeycomb size, the sandwich structure’s stiffness and stability are enhanced. However, the height of the honeycomb core has little effect on the impact response of the sandwich panel. Audibert [13] created a discrete model of the low-velocity impact on the Nomex honeycomb sandwich structure with CFRP face sheets. This discrete model strikes a fair balance between the mesoscopic representation of damage and the low computation time of the 3D continuous model. Additionally, experiments demonstrate the model’s effectiveness. Ivanez [14] studied the dynamic three-point bending behavior of composite sandwich beams through experiments and simulations. The failure mechanism of composite sandwich beams was investigated. At lower impact energies, the sandwich beam absorbed less impact energy and the aluminum core deformed, but the face sheet showed no evident damage. When the impact energy surpasses a particular threshold, the upper face sheet fails, and the sandwich beam absorbs the majority of the impact energy. Din [15] studied the effect of loading rate on the piezoresistive properties of sandwich structures. The results show that the piezoresistivity of the fabric sensor is inversely proportional to the loading rate. Changing the type of face sheet will make the structure have different mechanical properties [16]. Shin [17] investigated the low-velocity impact response of four sandwich structures utilized for low-floor buses in Korea. According to studies, the impact resistance of sandwich panels with woven glass fabric/epoxy face sheets is greater than that of structures with aluminum face sheets. Langdon [18] examined the response to blast loads of sandwich panels with different core heights and different skins. The results reveal that the residual deformation of the panels with composite face sheets is less than that of aluminum face sheets. Therefore, sandwich structures with composite face sheets have superior impact resistance.

Changing the structure of the honeycomb core is also an important method to improve the mechanical properties of the sandwich structure [19]. The standard honeycomb is a two-dimensional (2D) structure made of connected unit cells. Compared with matrix materials, honeycombs have higher porosity and lower mass density, resulting in higher specific stiffness, specific strength and specific energy absorption [20]. The unit cell has a significant effect on the overall mechanical properties [21]. Therefore, it is of utmost importance to impart extra mechanical properties to the honeycomb through sensible structural design. Among the different honeycomb structures, the negative Poisson’s ratio honeycomb structure has garnered interest due to its multiple engineering and practical properties [22,23]. Enhanced impact resistance is one of the most notable features [24]. As a result, honeycombs with a negative Poisson’s ratio (NPR) have superior performance when used as the core of sandwich structures. Najaf [25] used the honeycomb structure as the core of the sandwich beam and studied the three-point bending performance of four kinds of honeycomb structures, including re-entrant honeycomb. The results show that choosing the proper core topology has a significant impact on the flexural performance of sandwich beams. Yang [26] compared the mechanical properties of aluminum foam sandwich panels and re-entrant honeycomb sandwich panels under high-speed impact. Due to the material shrinkage in the impact area of the re-entrant honeycomb sandwich panel generated by the NPR effect, the bulletproof performance of the re-entrant honeycomb sandwich panel is significantly superior to that of the aluminum foam sandwich panel. Qi [27] examined the local impact performance of the re-entrant honeycomb sandwich structure and discovered that the re-entrant honeycomb has more resistance to impact than the traditional honeycomb. Hou [28] investigated the dependability of different structures by employing dynamic three-point bending tests on auxetic and non-auxetic lattice composites. It was discovered that the re-entrant honeycomb structure has a limited energy absorption capacity but is exceptionally resistant and durable. Therefore, the impact resistance of the re-entrant honeycomb is exceptional. As a typical honeycomb with an NPR, the re-entrant honeycomb has a straightforward construction and high designability. However, the majority of the present study is concentrated on the in-plane direction of the re-entrant honeycomb, and its capability in the out-of-plane direction still requires improvement and investigation. In addition, Li [29] designed, modeled and analyzed a cylindrical sandwich shell with a 3D double-v-element lattice core, revealing its nonlinear dynamic response when subjected to low-velocity impact. At the same time, Li [30] proposed an auxetic 3D lattice core and studied the impact resistance of its sandwich beam structure through experiments and simulations. It is found that the increased auxetic 3D lattice core can provide better resistance than the re-entrant honeycomb core with the same relative density.

With advancements in science and technology, people have understood that studying and imitating biological models or systems is one of the most important approaches to creating new technologies [31]. By analyzing biological models and emulating the internal model, complex engineering challenges can be solved. The study of high-performance structures based on bio-inspired design is ongoing [32,33]. In recent years, inspired by natural materials, the integration of biological models with honeycombs to create high-performance honeycombs has also been developed gradually. Zhang [34] presented a novel sort of honeycomb structure as a new type of lightweight energy absorber, that was inspired by bionic structures such as spider webs. Yang [35] combined a structure resembling petals with a circular honeycomb and proposed a novel circular honeycomb structure. Numerical analysis was used to examine the in-plane crushing behavior and energy absorption capacity of a honeycomb with a petal-like form subjected to varying impact velocities. Under the same impact velocity, the results indicate that the specific energy absorption of the improved structure is nearly two times that of the standard circular honeycomb. Zhang [36] proposed a re-entrant arc-shaped honeycomb model based on the design idea of a bionic structure to improve the impact energy absorption capacity of special-shaped honeycombs and maintain good uniformity of the crushing load. The in-plane impact resistance and energy absorption characteristics of bionic toughened billets under constant velocity crushing conditions were explored using numerical modeling. Under the condition of achieving the lightweight requirements, the mechanical properties of the honeycomb structure inspired by the biological model have been greatly enhanced, thereby expanding the bionic honeycomb’s application range [37,38,39]. However, there is currently a paucity of research aimed at enhancing the out-of-plane features of re-entrant cells. Consequently, it is still important to enhance the mechanical properties of the structure based on a re-entrant honeycomb. From the preceding, it can be found that the honeycomb-like core of the sandwich structure is often a 2D structure. Compared to the 2D structure, a three-dimensional (3D) auxetic structure can exhibit the NRP effect in more directions and yield better stiffness and strength increases under the influence of a local impact load, making it more attractive for impact-resistant applications. Most existing 3D auxetic structures are trusses, which contain many discontinuous gaps and may be weak in specific areas under load. To solve these issues, it is possible to create a 3D honeycomb-like NPR structure.

Observing and imitating biological organisms is frequently practiced by humans to create new technologies and instruments. For instance, ancient craftsmen created fish scale armor by observing the arrangement of fish scales. Because the mosaic arrangement of fish scales disperses received force and protects the fish’s body while allowing it to move freely, this benefit of fish scales is also well transmitted to fish scale armor. In addition to fish, several other animals possess scales. The existence of such scales also protects the animals themselves, which motivates craftsmen around the globe to invent similar structures by studying animals. The majority of scales on a living body are grouped in a staggered pattern and also have a spatial structure.

Re-entrant honeycombs conform to this staggered arrangement, but the traditional NPR honeycomb is a 2D structure constructed by stretching the honeycombs in the 2D plane along the out-of-plane direction. This staggered arrangement occurs only inside the same plane, and the force cannot be dispersed effectively when the load is outside the plane. Therefore, referring to the staggered arrangement of fish scales in space, the 2D re-entrant honeycomb is improved. In order to further improve the impact resistance of the sandwich structure, a 3D re-entrant honeycomb unit and a honeycomb stacking method are proposed in this study. The resultant novel re-entrant honeycomb was utilized as the core of the sandwich structure in order to improve the impact resistance of the sandwich structure under impact loads. The proposed structure not only satisfies the lightweight requirement but also increases stiffness and strength under local impact loads, making it more suitable for use in impact-resistant conditions. Therefore, it is expected that the research on this three-dimensional re-entrant honeycomb sandwich structure would not only be applicable to the working circumstances of impact resistance but also provide a new design reference for honeycomb sandwich structure in the future.

This study aims to investigate the mechanical properties of honeycomb sandwich structures under low-velocity impact loads using experimental and numerical methods. The core of the sandwich structure is made by 3D printing. By means of low-velocity impact tests, the mechanical properties of the structure under different impact energies were studied. In order to further explore the influence of structural parameters on the structural, mechanical properties, a numerical model was established based on Abaqus, and the validity of the model was verified by experiments. The influence of the structural parameters on the peak crushing force, contact time and energy absorption was explored by numerical models.

## 2. Theory and Design

**Figure 1:**
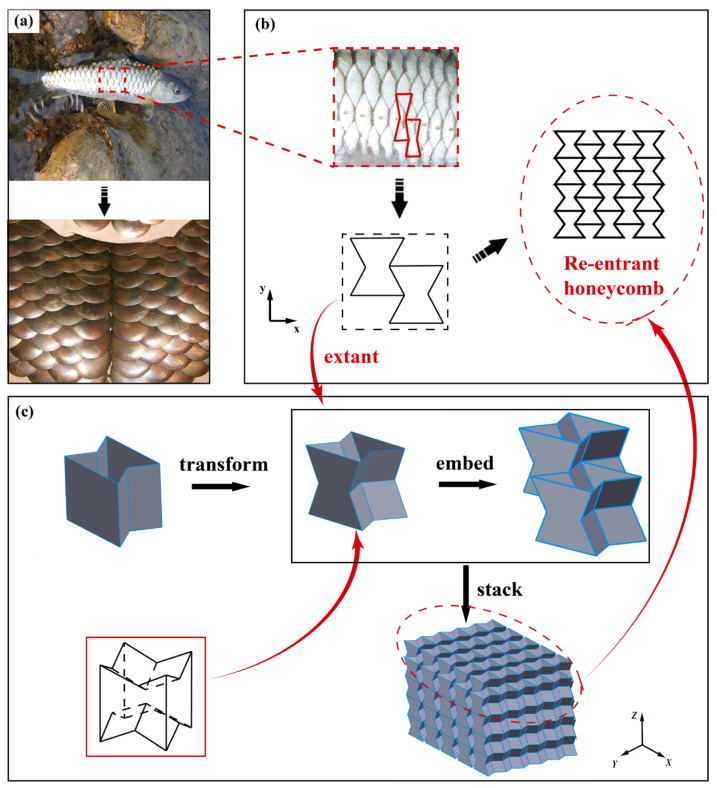
Re-entrant honeycomb unit cell design. (**a**) fish scale armor; (**b**) 2D re-entrant honeycomb; (**c**) 3D re-entrant honeycomb.

The distinctive arrangement of fish scales protects the fish’s body and enables it to move freely. Additionally, craftsmen invented fish scale armor inspired by fish scales. The whole fish scales can be regarded as formed by a periodic arrangement of re-entrant honeycombs. However, the conventional re-entrant honeycomb is a 2D construction. The 2D re-entrant honeycomb is thereby extended into a 3D structure. Referring to the arrangement of fish scales, the 2D re-entrant honeycomb has been improved. The straight side walls of the honeycomb are concave to form a structure similar to an eggshell frame, which is used to transmit the side force. The structure is seen in Figure 1. Unlike the straight walls of traditional honeycombs, the unit cells of the enhanced honeycomb are simpler to embed when stacked. Due to the material characteristics and self-locking effect induced by the unit-cell stacking approach, this newly built structure not only functions as an energy-absorbing device but also has sufficient rigidity.

**Figure 2:**
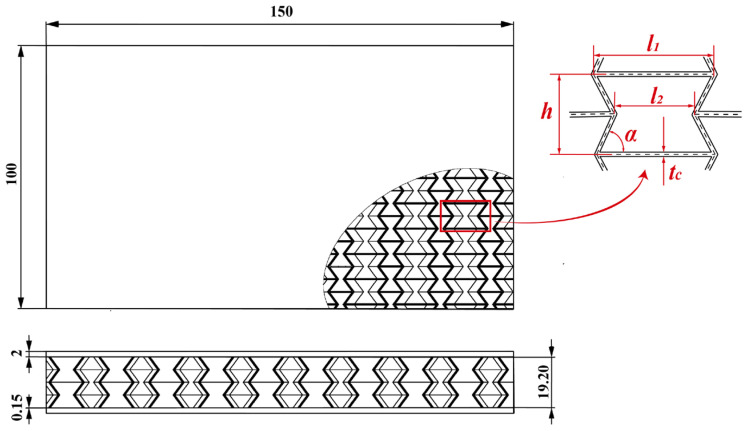
The schematic of the re-entering honeycomb sandwich panel.

To ensure that there is no collision between the faces of the unit cell during the folding process of the structure, the unit cell’s overall spatial design is similar to a cube. Concurrently, the size of the concave hexagon on each of the four faces of the unit cell is maintained in order to prevent the experimental deviation produced by the uneven size. The structure of the re-entering honeycomb sandwich panel is depicted in Figure 2. Among them, the longer side of the unit cell is $l_{1}$ = 14 mm, the distance between the concave points on both sides is $l_{2}$ = 8 mm, the height is h = 9.6 mm, the concave angle is α = 58°, and the wall thickness of the unit cell is $t_{c}$ = 0.5 mm.

## 3. Experiments and Simulation

### 3.1. Specimen and Materials

**Figure 3:**
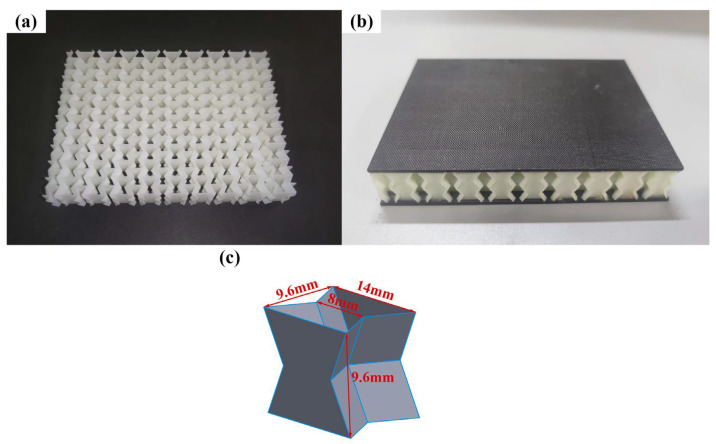
Re-entrant honeycomb sandwich panel. (**a**) core; (**b**) specimen; (**c**) a unit cell.

Due to the limitations of the experimental equipment, the re-entrant honeycomb core employs only two layers of unit cells to lower the structure’s height. The honeycomb core was made using SLA technology. Godart^®^8228 is a photosensitive resin produced by GreatSimple Technology Inc., (Zhongshan, China). After forming, the material density is 1180 kg/m^3^. The honeycomb specimen is depicted in Figure 3a. Three specimens were printed, each measuring 19.2 mm in height and 0.5 mm in wall thickness. The relative density of the honeycomb core is 0.1421. The experimental specimens were prepared to the ASTM D7766/D7766M-11 standard [40]. To improve the impact resistance of the face sheet, CFRP panels are utilized. The CFRP panel produced by China’s Weihai Guangwei Composite Material Co., Ltd. has a carbon fiber model of T300 and a matrix model of 7901. The matrix is an epoxy resin. The CFRP panel has a [0/90]_5_ layering method, a total thickness of 2 mm, and a single-layer thickness of 0.2 mm. The specific mechanical parameters of the T300/7901 laminates are shown in Table 1 [41]. The adhesive layer between the face sheet and the core is made of ductile epoxy resin Araldite 2015 and has a nominal thickness of 0.15 mm. All of the specimens consisted of face sheets and cores bonded by room-temperature curing epoxy resin Araldite 2015. Araldite 2015 is a two-component, room-temperature-curing paste adhesive. In order to make the core and the face sheet more tightly bonded and to prevent the core from plastic deformation due to excessive pressure, a pressure of 1 KPa is applied to the surface of the specimen. Considering the change in room temperature, the sandwich panels were cured for 72 h at room temperature to achieve satisfactory adhesion [12]. The material properties of the core and adhesive refer to [19], and the parameters are shown in Table 2. The specimen is shown in Figure 3.

### 3.2. Low-Velocity Impact Experiment

**Figure 4:**
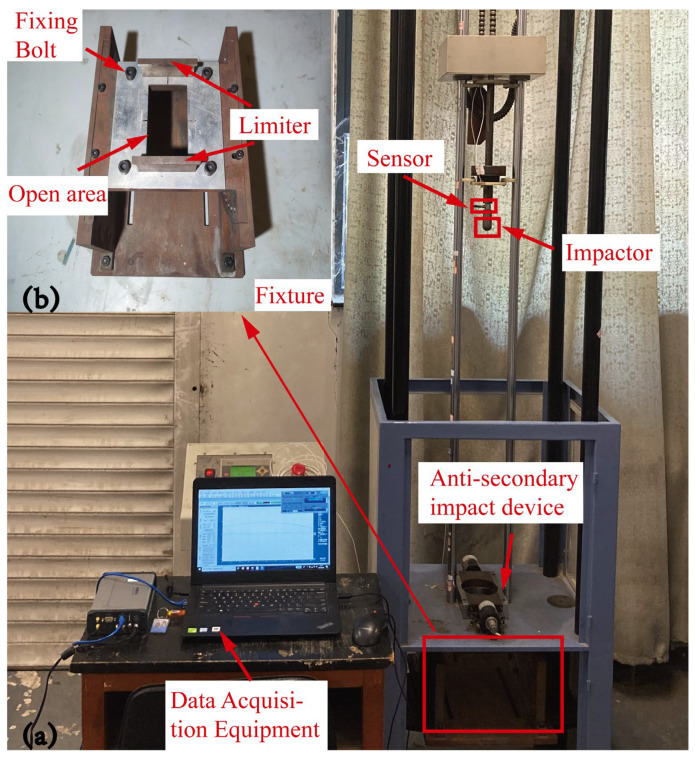
Drop-weight test system. (**a**) Impact test platform, (**b**) Fixture.

A low-velocity impact experiment was designed according to the ASTM D7136M standard [42]. The sample’s base is supported by a steel plate with an open area. Additionally, another steel plate with an open area is placed on top of the specimen in order to clamp it. As depicted in Figure 4, the impact experiment is carried out using the WLJ 300 drop-weight impact test system, which includes a drop-weight tower, a control platform, a data gathering system and an anti-secondary impact device. The impactor’s cap is hemispherical. It has a 25 mm diameter and weighs 2.5 kg. By adjusting the impactor’s height, various impact energies can be achieved. During the descending phase of the impactor, frictional energy loss is disregarded. Three groups of studies were conducted under 10 J, 20 J and 30 J impact energies, respectively, in order to compare the mechanical properties of the sandwich panels under different energies.

### 3.3. Finite Element Model

**Figure 5:**
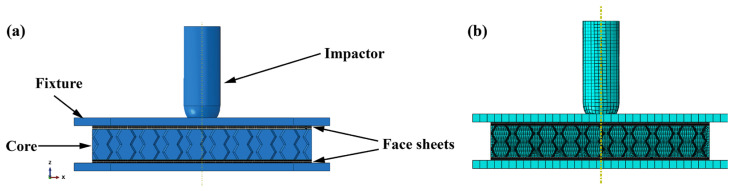
The finite element model. (**a**) 3D model, (**b**) model with mesh.

The geometric model was constructed with Abaqus/Explicit using the aforementioned experiments as a guide. In the simulation, the model represented in Figure 5 is established. All of the models in the simulation are constructed according to the experiment’s geometric dimensions. The top of the impactor is hemispherical, with a mass of 2.5 kg and a diameter of 25 mm. As rigid bodies, both the impactor and the fixture are restricted. The impactor has only one degree of freedom on the z-axis, while the fixture restricts all degrees of freedom. The impactor and fixture are composed of C3D8R elements. Before numerical calculation, the impactor is given the initial velocity corresponding to the impact energy. The specimen was fixed by two upper and lower steel plates according to the experiment. Additionally, the steel plates are fixed. To imitate delamination damage, the face sheet is discretized using C3D8R solid elements, and each interlayer adhesive is introduced with 0-thickness COH3D8 elements. The mesh size of the face sheet and the interlayer adhesive is 1.5 mm. The 3D Hashin criterion was utilized to evaluate the damage within the damaged CFRP face sheets, and the cohesive force unit was used to evaluate the delamination damage of the composite laminate’s interlayer adhesive [41,43]. The 3D Hashing criterion was used to predict the progressive failure of the composite laminates after the initial repair. On this basis, the residual strength of the damaged laminate was calculated using the continuum damage model (CDM). The cohesive zone model (CZM) was used to describe the interlayer damage of the face sheets. The CZM method follows the law of traction separation. Before peeling and delamination, the constitutive relation is considered linear elastic. Similar to intralaminar damage, the damage evolution of interlaminar damage can be divided into two steps. The interactions are considered to have linear elastic behavior until delamination and debonding. Once the damage criterion is met, the adhesion decreases linearly. The core of the re-entrant honeycomb is comprised of S4R shell elements. The mesh size of the honeycomb is 1 mm. The honeycomb was assumed to be an elastoplastic material. Table 2 shows the mechanical properties. Consequently, the adhesive is simulated using an elastoplastic model with reference to the pertinent parameters in [19]. Table 2 [19] lists the specific parameters of the core and adhesive. The surface-to-surface contact property is set between the impactor and the upper face sheet, and its normal direction adopts the “hard” contact model. Adjust the friction coefficient to 0.3 [41]. To avoid element penetration, a generic contact model is employed between additional components, with the same normal and tangential contact qualities as described above.

## 4. Results and Discussion

### 4.1. Validation of Numerical Models

**Figure 6:**
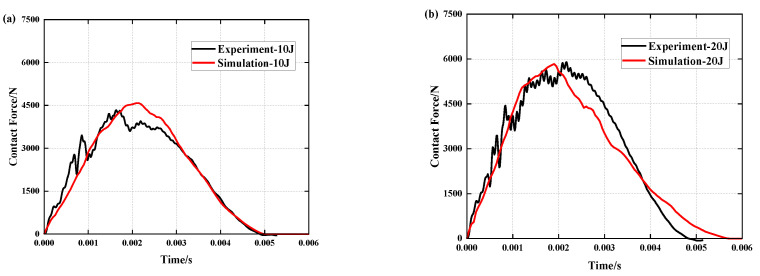

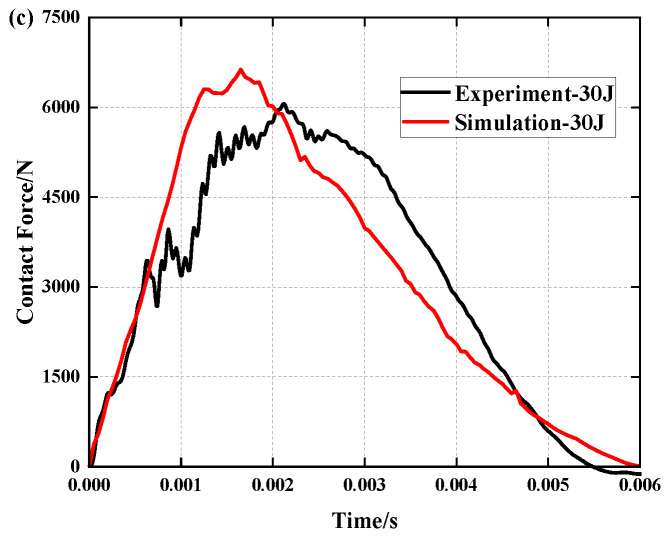
Comparison of simulation and experiment contact force histories under different impact energies: (**a**) 10 J, (**b**) 20 J and (**c**) 30 J.

Figure 6 depicts the results of a comparison of simulation and experiment contact force histories under different impact energies, while Table 3 and Table 4 compare simulation and experiment contact force and contact time data. By comparing the data, it can be determined that under the influence of three distinct impact energies, the deviation between simulation and experiment for the peak contact force is within 10%. Simulation and experiment differ in contact time by 0.054 ms, 0.852 ms and 0.516 ms, respectively. The curves of the experiment and simulation can be well fitted. During the experiment, however, due to the unpredictability of the friction between the impactor platform and the slide rail and the impact of external vibration on the equipment, the contact force fluctuated, or the peak value of the force produced errors so that the experimental curve and the simulation curve could not coincide exactly.

**Figure 7:**
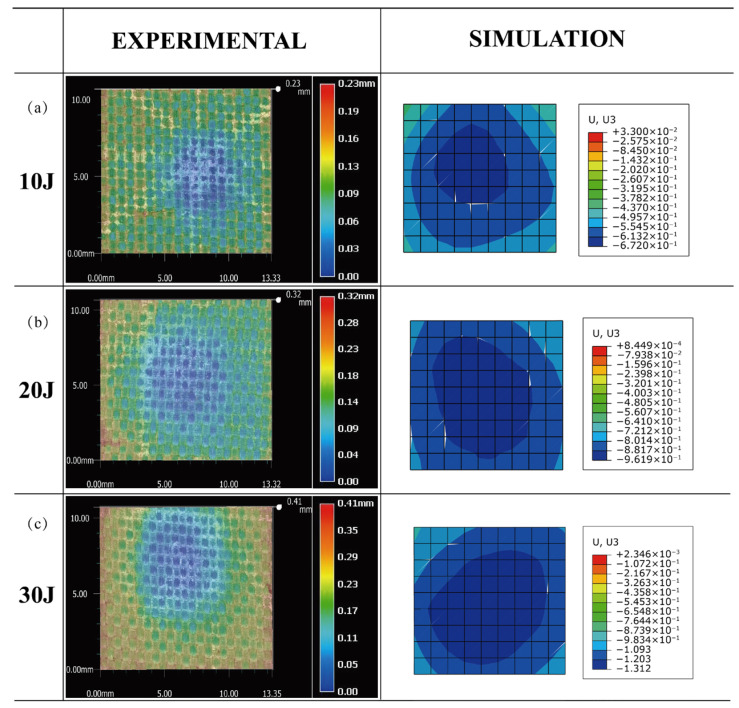
Comparison of the maximum deformation between the experiment and simulation of the honeycomb sandwich panel. (**a**) 10 J, (**b**) 20 J and (**c**) 30 J.

After the experiment, the surface of the specimen exhibited no obvious damage. At the impact area, only minor dents were seen on the specimen’s upper face sheet. Figure 7 depicts a comparison of the maximum deformation between the experiment and simulation of the honeycomb sandwich panel subjected to three different impact energies in order to further validate the model’s accuracy. Using an Ultra-Depth Three-Dimensional Microscope Keyence VHX-6000, the impact area was observed and compared to the simulation. The deformation depths achieved by simulation under different impact energies are 0.235 mm, 0.321 mm and 0.438 mm, while the deformation depths of the corresponding impact areas acquired by the experiment are 0.23 mm, 0.32 mm and 0.41 mm. Observably, the experimental and modeled maximum deformations are similar. In conclusion, the simulation model developed in this study may more accurately forecast the mechanical properties of sandwich panels subject to impact loads.

### 4.2. Mechanical Properties Comparison with Traditional Re-Entrant Honeycomb

**Figure 8:**
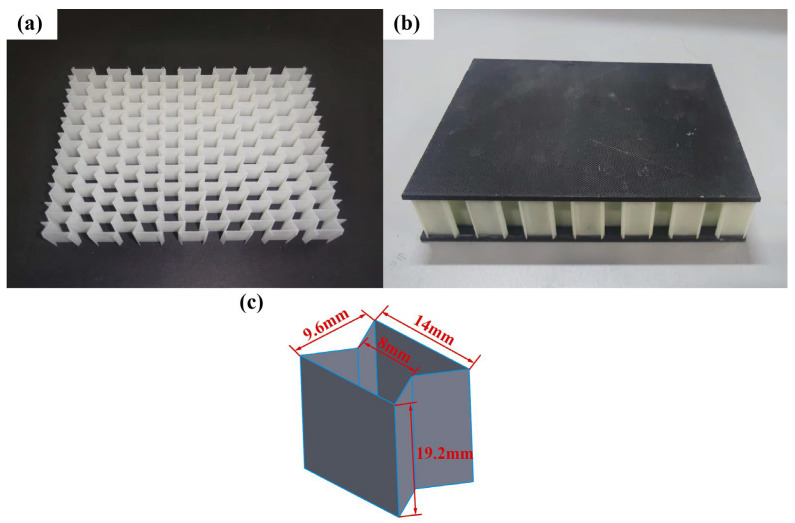
Traditional re-entrant honeycomb sandwich panel. (**a**) core; (**b**) specimen; (**c**) a unit cell.

To study the mechanical properties of the enhanced honeycomb sandwich panel, it was compared to a standard re-entrant honeycomb structure. Traditional re-entrant honeycomb sandwich panels were subjected to low-velocity impact experiments under the same experimental conditions as those described before. The traditional re-entrant honeycomb sandwich panel is different from the improved sandwich panel only in the honeycomb core. The traditional re-entrant honeycomb has the same concave hexagon size as the improved honeycomb. To ensure that both structures have the same relative density, the thickness of the traditional structure’s walls is set to 0.6 mm, while all other conditions remain unchanged. The traditional re-entrant honeycomb sandwich panel is shown in Figure 8.

**Figure 9:**
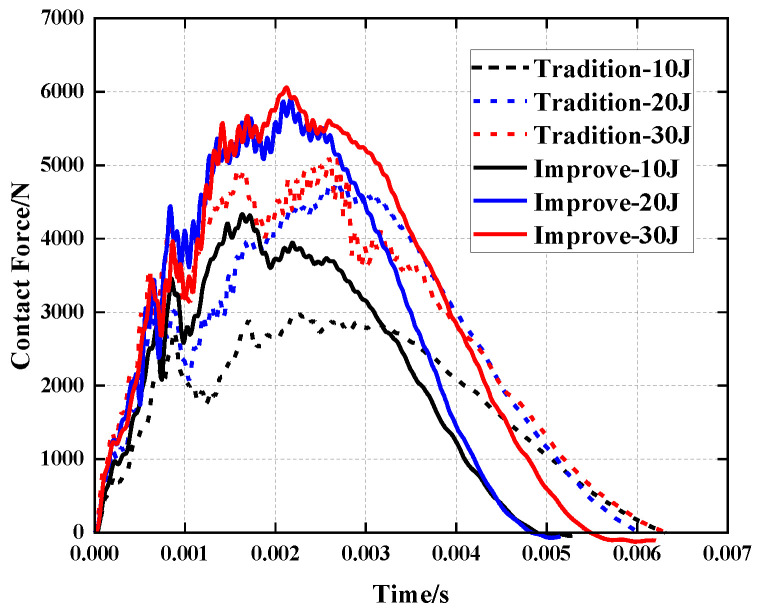
Comparison of contact force history of re-entrant honeycomb sandwich panels before and after improvement.

Figure 9 and Table 5 demonstrate that under the impact energy of 10 J, the peak force of the improved structure is 45.64% greater than that of the traditional re-entrant honeycomb sandwich panel, but the deformation of the face sheet is reduced by 14.82%. Under impact energies of 20 J and 30 J, the peak force rose by 23.15% and 17.14%, whereas the deformation depth of the face sheet decreased by 8.57% and 12.7%, respectively. The improved concave honeycomb provides a larger contact force at the same impact energy, less impact damage and enhanced resistance to impact.

### 4.3. Effects of Geometric Parameters

#### 4.3.1. Face Sheet Thickness

**Figure 10:**
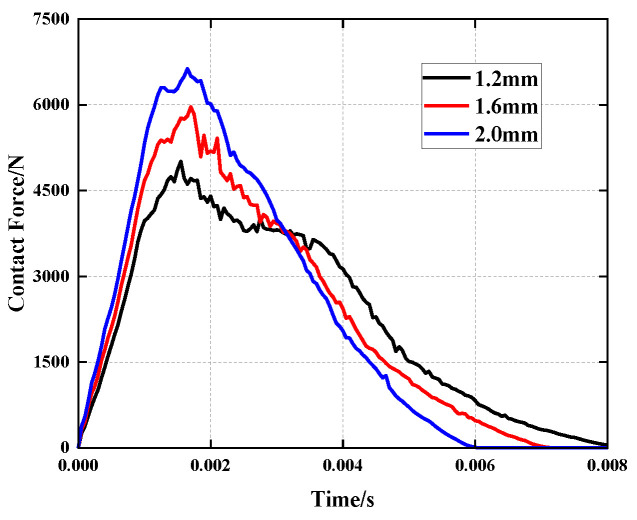
Comparison of the contact force history of the impactor under different face sheet thicknesses.

To examine the effect of face sheet thickness on the mechanical properties of the improved honeycomb sandwich panel when subjected to a low-velocity impact load, three face sheet thicknesses of $t_{c}$ = 1.2 mm, 1.6 mm and 2.0 mm were utilized in the simulation, while all of the other parameters were kept constant. As illustrated in Figure 10, as the thickness of the face sheet increases, so do the peak contact force, the contact time and the structural stiffness. Under 30 J of impact energy, the thickness of the face sheet increases by 0.8 mm and the peak contact force of the structure increases by 32.39%. Therefore, an increase in the thickness of the face sheet will increase the honeycomb sandwich panel’s rigidity, as well as the structure’s resistance to impact.

**Figure 11:**
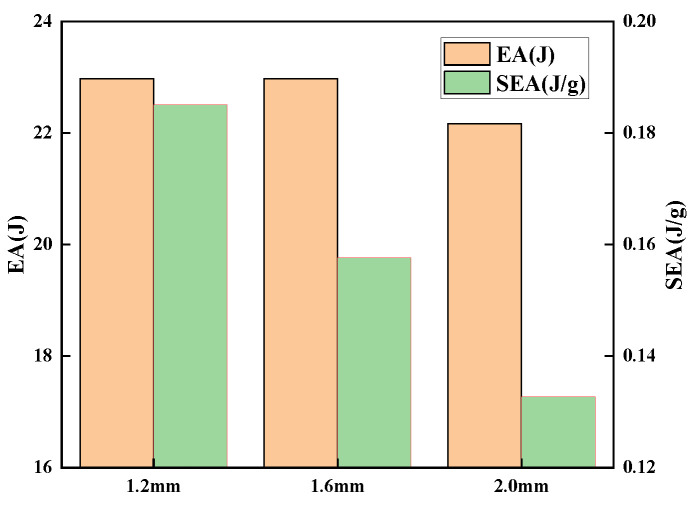
EA and SEA of sandwich panels with different face sheet thicknesses.

Figure 11 demonstrates the energy absorption and specific energy absorption of sandwich panels with various face sheet thicknesses. The energy absorption characteristics of the overall structure tend to first increase and then decrease as the thickness of the skin grows, but the range of change is modest. The specific energy absorption of the structure decreases rapidly, and the specific energy absorption decreases by 28.31% when the face sheet thickness increases by 0.8 mm. The face sheet that is too thick will raise the structure’s rigidity, which is not conducive to the deformation of the core, hence decreasing the structure’s energy absorption. However, if the face sheet is excessively thin, structural stiffness and impact resistance would be compromised.

#### 4.3.2. Concave Angle α

To investigate the effect of the concave angle on the mechanical properties of the structure when subjected to low-velocity impact loads, the size of the concave angle of the unit cell was altered in the simulation, while all other parameters remained unchanged. For calculation considerations, the size of the concave angle α is modified here by adjusting the size of $l_{2}$. Since too small concave angles will cause interference between cell surfaces, three types of unit cells with $l_{2}$ = 8 mm, 10 mm and 12 mm are chosen, and their concave angles correspond to 58°, 67° and 78°, respectively.

**Figure 12:**
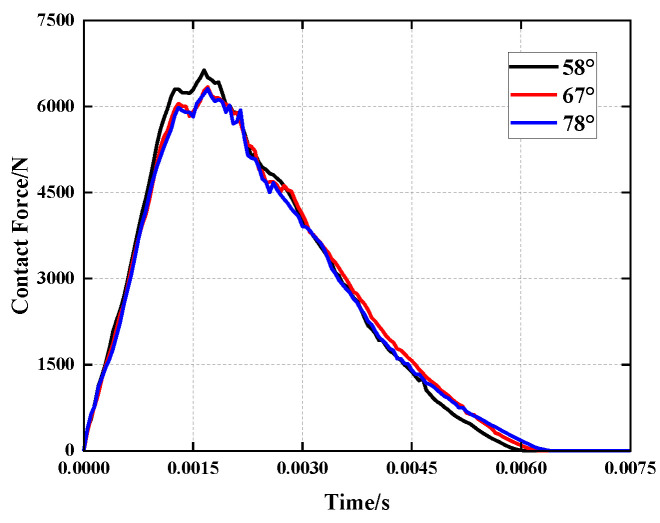
Comparison of the contact force history of the impactor under different concave angles.

**Figure 13:**
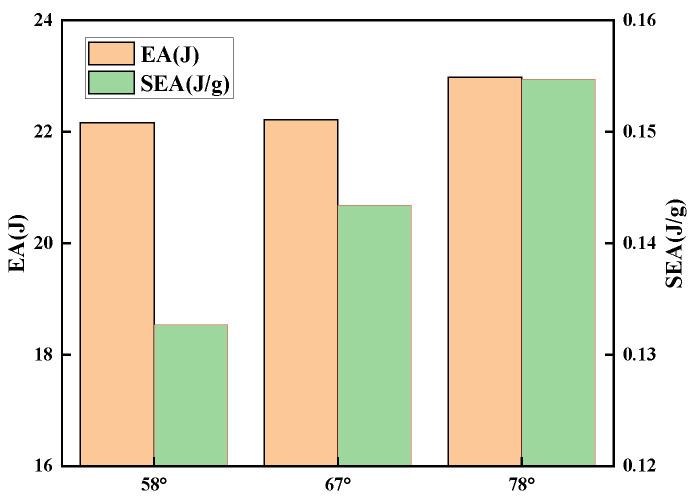
EA and SEA of sandwich panels with different concave angles.

Figure 12 and Figure 13 illustrate that when the concave angle grows, the simulation curve trend does not alter considerably, and the peak force decreases by less than 5%. When loaded, the particular stacking method of the unit cells causes the independent unit cells to distribute force to nearby units. In addition, due to the presence of side wall folding creases, the structure will initially flex along the folding creases as the load is applied. However, due to the connection of the unit cells, the unit cells’ deformation is impeded, and the total stiffness of the structure rises. Changing the concave angle has no effect on the structural deformation process under load. Changing the size of the concave angle in the honeycomb core does not affect the impact resistance of the sandwich panel. However, increasing the concave angle will increase the intercellular space ratio and decrease the relative density. This causes the structure to contract more slowly, approaching the impact area and reduces the effect of NPR. This modification will also enhance the overall energy absorption of the structure, as well as the specific energy absorption. Consequently, raising the concave angle can substantially increase the energy absorption characteristics of the structure while preserving its initial impact resistance.

## 5. Conclusions

In this study, the traditional re-entrant honeycomb sandwich panel is enhanced by referencing the arrangement of fish scales, and the impact resistance of the structure before and after the improvement is compared under low-velocity impact. To further investigate the effect of structural parameters on the mechanical properties of sandwich panels, a low-velocity impact simulation model was developed, and the mechanical properties of sandwich panels subjected to low-velocity impact loads were studied.

The study indicated that the proposed improved structure offers superior impact resistance compared to traditional re-entrant honeycomb sandwich panels, as well as stronger contact forces and less deformation when subjected to impact loads. When the sandwich panel is impacted by low energy, small depressions occur on the upper face sheet of the sandwich panel. Compared with the traditional structure, the concave depth of the face sheet on the improved structure is smaller, and the damage depth is reduced by an average of 12%. Under an impact energy of 10 J, the improvement of the core increased the peak force of the sandwich panel by 45.64%. Additionally, the 3D re-entrant honeycomb can be utilized as an energy-absorbing device, and due to the unevenness of the cell wall and the stacking of cells, the structure has a certain degree of stiffness when loaded. Increasing the thickness of the face sheet of the sandwich panel will improve the impact resistance of the structure, but an excessively thick face sheet will increase the rigidity of the structure, which is not conducive to the deformation of the core, resulting in a reduction in the energy absorption of the structure and a decline in the overall quality. The expansion causes the structure to diminish quicker than it can absorb energy. According to the study, the magnitude of the concave angle has a minimal effect on the impact resistance of the sandwich panel, and reducing the concave angle does not significantly increase the structure’s impact resistance. Nevertheless, increasing the concave angle will increase the structural-specific energy absorption of the core, assuming the structure remains unchanged.

## Figures and Tables

**Table 1:** Mechanical parameters of T300/7901 laminates [41].

Properties	T300/7901	Properties	Inter-Laminar Adhesive
$E_{1}/\mathrm{MPa}$	125,000	$G_{n}^{C}/(N\cdot mm^{-1})$	0.52
$E_{2},E_{3}/\mathrm{MPa}$	11,300	$G_{s}^{C},G_{t}^{C}/(N\cdot mm^{-1})$	0.92
$G_{12},G_{13}/\mathrm{MPa}$	5430	${\sigma /\mathrm{MPa}}_{n,max}$	50
$G_{23}/\mathrm{MPa}$	3979	${\sigma /\mathrm{MPa}}_{s,max}$	94
$\nu_{12},\nu_{13}$	0.3	${\sigma /\mathrm{MPa}}_{t,max}$	94
$\nu_{23}$	0.42	$K_{n}/(N\cdot mm^{-3})$	100,000
$X_{T}/\mathrm{MPa}$	2000	$K_{s},K_{t}/(N\cdot mm^{-3})$	100,000
$X_{C}/\mathrm{MPa}$	1100		
$Y_{T},Z_{T}/\mathrm{MPa}$	80		
$Y_{C},Z_{C}/\mathrm{MPa}$	280		
S/MPa	120		

($E_{i}(i=1,2,3)$ is the Young’s modulus in the i direction; $G_{ij}(i,j=1,2,3)$ is the shear modulus of plane i−j; $v_{ij}\left(i,j=1,2,3\right)$ is the Poisson’s ratio of plane i−j; $X_{T}$ and $X_{C}$ are the tensile strength and compressive strength in the 1 direction, respectively; $Y_{T}$ and $Y_{C}$ are the tensile strength and compressive strength in 2 directions, respectively; S is the shear strength; $G_{n}^{C}$ is the tensile toughness; $G_{s}^{C}$ and $G_{t}^{C}$ are the shear toughness components in the 1-direction and 2-direction, respectively; $\sigma_{n,max}$ is the maximum nominal stress in the normal-only mode; $\sigma_{s,max}$ and $\sigma_{t,max}$ are the 1-direction and 2-direction The maximum nominal stress in the direction; $K_{n}$ is the tensile stiffness; $K_{s}$ and $K_{t}$ are the shear stiffness components).

**Table 2:** Material parameters of core and adhesive [19].

Materials	Density (kg/m^3^)	Young’s Modulus (GPa)	Poisson’s Ratio	Yield Stress (MPa)	Tensile Strength (MPa)	Failure Strain
Godart ^®^8228	1180	2.1	0.35	-	51.21	0.16
Araldite 2015	1400	1.85	0.33	12.63	21.63	0.047

**Table 3:** Comparison of peak contact force in experiment and simulation under different impact energies.

Energy/J	Experimental Value/N	Simulation Value/N	Deviation/N	Relative Error/%
10	4335.57	4584.32	248.75	5.74
20	5897.92	5832.76	65.16	1.10
30	6058.96	6632.77	573.81	9.47

**Table 4:** Comparison of contact time in experiment and simulation under different impact energies.

Energy/J	Experimental Value/ms	Simulation Value/ms	Deviation/ms
10	4.896	4.95	0.054
20	4.848	5.70	0.852
30	5.484	6.00	0.516

**Table 5:** Comparison of face sheet deformation depth of two structures under different impact energies.

Energy/J	Traditional Structures/mm	Improved Structure/mm
10	0.27	0.23
20	0.35	0.32
30	0.47	0.41

## Data Availability

Data presented in this study are available upon request from the corresponding author.
